# Association of Donor and Recipient Telomere Length with Clinical Outcomes following Lung Transplantation

**DOI:** 10.1371/journal.pone.0162409

**Published:** 2016-09-02

**Authors:** Andrew M. Courtwright, Sabrina Fried, Julian A. Villalba, Anna Moniodis, Indira Guleria, Isabelle Wood, Edgar Milford, Hari H. Mallidi, Gary M. Hunninghake, Benjamin A. Raby, Suneet Agarwal, Philip C. Camp, Ivan O. Rosas, Hilary J. Goldberg, Souheil El-Chemaly

**Affiliations:** 1 Division of Pulmonary and Critical Care, Brigham and Women’s Hospital, Boston, Massachusetts, United States of America; 2 Tissue Typing Laboratory, Brigham and Women’s Hospital, Boston, Massachusetts, United States of America; 3 Division of Thoracic Surgery, Brigham and Women’s Hospital, Boston, Massachusetts, United States of America; 4 Channing Division of Network Medicine, Brigham and Women’s Hospital, Boston, Massachusetts, United States of America; 5 Division of Hematology/Oncology, Boston Children’s Hospital, Boston, Massachusetts, United States of America; University of Toledo, UNITED STATES

## Abstract

**Background:**

Patients with short telomere syndromes and pulmonary fibrosis have increased complications after lung transplant. However, the more general impact of donor and recipient telomere length in lung transplant has not been well characterized.

**Methods:**

This was an observational cohort study of patients who received lung transplant at a single center between January 1^st^ 2012 and January 31^st^ 2015. Relative donor lymphocyte telomere length was measured and classified into long (third tertile) and short (other tertiles). Relative recipient lung telomere length was measured and classified into short (first tertile) and long (other tertiles). Outcome data included survival, need for modification of immunosuppression, liver or kidney injury, cytomegalovirus reactivation, and acute rejection.

**Results:**

Recipient lung tissue telomere lengths were measured for 54 of the 79 patients (68.3%) who underwent transplant during the study period. Donor lymphocyte telomeres were measured for 45 (83.3%) of these recipients. Neither long donor telomere length (hazard ratio [HR] = 0.58, 95% confidence interval [CI], 0.12–2.85, p = 0.50) nor short recipient telomere length (HR = 1.01, 95% CI = 0.50–2.05, p = 0.96) were associated with adjusted survival following lung transplant. Recipients with short telomeres were less likely to have acute cellular rejection (23.5% vs. 58.8%, p = 0.02) but were not more likely to have other organ dysfunction.

**Conclusions:**

In this small cohort, neither long donor lymphocyte telomeres nor short recipient lung tissue telomeres were associated with adjusted survival after lung transplantation. Larger studies are needed to confirm these findings.

## Introduction

Telomeres are repetitive nucleotide sequences at the ends of chromosomes that become truncated with successive cellular replication. Telomere shortening can trigger cellular senescence and apoptosis. Loss of function mutations in telomere maintenance pathways are associated with premature organ dysfunction including bone marrow failure, cirrhosis, enteropathies, and interstitial lung disease[1]. Short telomeres have also been associated with other pulmonary diseases such as emphysema, although whether this is a consequence of smoking-mediated telomere shortening or a genetic predisposition to telomere shortening that manifests as emphysema in some smokers remains poorly understood [2–4]. Similarly almost 30% of patients with idiopathic pulmonary fibrosis (IPF) have short telomeres with or without telomerase mutations[5].

For patients with advanced IPF associated with short telomere syndromes, lung transplantation is the only definitive treatment. These individuals, however, are at risk for worse outcomes following transplant both because they are more susceptible to bone marrow suppressive effects of anti-rejection medication and because of reduced extra-pulmonary organ reserve [6, 7]. For example, Silhan et al found that patients with telomerase mutations and IPF were more likely to require platelet transfusions, to need dialysis, and to have adjustment of immunosuppressives following transplant[8]. Similarly, Borie et al found a high rate of bone marrow failure and death following lung transplant in patients with telomerase mutations[9].

Outside of patients with known short telomere syndrome, however, there are few data on telomere length in lung transplantation from either the donor or recipient standpoint. It is unknown what portions of donors and recipients have telomeres significantly below population means. It is also unknown whether shorter recipient telomeres, in general, are associated with need for modification of immunosuppression, extra-pulmonary organ dysfunction, or worse survival after lung transplant. Theoretically, these patients may have decreased extra-pulmonary reserve, even in the absence of a known telomere shortening mutation, which may manifest as similar complications following transplant.

From the donor standpoint, longer donor telomere length might provide increased pulmonary reserve following transplant, improving survival and allograft function. Supporting this idea, among hematopoietic cell transplant (HCT) recipients with aplastic anemia, longer donor telomere length is associated with improved recipient survival[10]. Although data are limited among patients with hematologic disorders other than aplastic anemia, given that donor telomeres undergo accelerated shortening following HCT, recipients with shorter donor telomeres may be at higher risk for relapse or late graft failure[11, 12].

The primary objective of this study was to characterize donor and recipient telomere length in lung transplant patients. Our secondary objective was to perform a preliminary assessment of the association of donor telomere length with survival following transplant and recipient telomere length with survival, acute rejection, and extra-pulmonary organ dysfunction. Our hypotheses were that longer donor telomere length would be correlated with improved survival and that shorter recipient telomere length would be associated with decreased survival and increased extra-pulmonary organ dysfunction.

## Materials and Methods

### Study population

The Partners Human Research Committee Brigham and Women’s Hospital Institutional Review Board approved this study. All patients who underwent lung transplantation at Brigham and Women’s Hospital from January 1st 2012 to January 31st 2015 were eligible to participate at the time of transplant. Written informed consent was obtained from patients who participated. Patients who did not provide informed consent and patients whose lung tissue was unavailable for testing were excluded.

### Donor telomere length measurement

We extracted genomic DNA from donor lymph nodes or spleen collected prior to organ procurement. Relative telomere length was determined using a high throughout monoplex quantitative real time polymerase chain reaction assay as previously described[13]. Briefly, this assay determined the ratio between telomeric repeat copy number (T) and a single copy reference gene, 36B4 (S). The T/S ratio was calculated by subtracting the average 36B4 threshold cycle value from the average telomere threshold cycle value. The relative T/S ratio was then calculated by subtracting the T/S ratio of a reference sample, consisting of a pooled genomic DNA sample, from the patient’s T/S ratio. In this way, relative T/S was defined in relation to a population reference curve with final measurements exponentiated to assure normality[14]. We divided donors into those with long (third tertile) and short (other tertiles) telomeres as previously described [9, 15].

### Recipient telomere length measurement

Pieces of explanted lungs were immediately snap frozen on dry ice and stored in liquid nitrogen. We extracted genomic DNA from a 1x1 cm^2^ sized specimen of the explanted whole lung tissue and determined relative telomere length in a similar fashion as donor lymphocyte telomere length. We were unable to isolate a specific type of lung tissue for this analysis and the reported recipient telomere length likely represented multiple tissue types. We divided recipients into those with short (first tertile) and long (other tertiles) telomeres.

### Outcomes and predictor variables

The primary outcome was survival following transplant. Follow-up time started at date of transplant and ended at death or on May 1^st^ 2016, the time of data analysis. The primary predictor variables of interest were long donor telomere length and short recipient telomere length. We also collected data on clinical and demographic characteristics that may impact survival following transplant including recipient and donor age, native lung disease (IPF vs. non-IPF), lung allocation score (LAS) at transplant, and most recent percent predicted forced expiratory volume in 1 second (FEV1).

We collected data on secondary outcomes during the first year following transplantation including: 1) leukopenia requiring granulocyte colony stimulating factor (GCSF) or cessation of immunosuppression or antiviral medication; 2) acute kidney injury defined as a rise in creatinine 1.5 times baseline over one week; 3) need for dialysis; or 4) liver injury, defined as alanine aminotransferase or aspartate aminotransferase great than three times the upper limit of normal (129 and 183 units per liter, respectively). Other secondary outcomes included cytomegalovirus (CMV) reactivation after stopping antiviral prophylaxis and acute cellular rejection (ACR) of any grade.

### Statistical analysis

We used descriptive statistics to identify percentages, medians and quartiles, and means and standard deviations for selected demographic and clinical variables. We used Pearson’s correlation coefficients to assess the relationship between donor age and donor telomere length and recipient age and recipient telomere length. We conducted two survival analyses using Cox proportional hazard models to determine the impact of: 1) long donor telomeres and 2) short recipient telomeres on survival following transplant. We included recipient and donor age, native lung disease, LAS, and most recent percent predicted FEV1 as covariates in both models. We examined Schoenfeld residuals to confirm the proportional hazard assumption. A joint donor and recipient telomere length analysis was not performed because of the small numbers of patients with donor-recipient length discordance.

We used Fisher exact tests to compare the above secondary outcomes for recipients with short versus long lung tissue telomeres with 2-sided p<0.05 considered statistically significant. All analyses were performed using Stata (Version 14, Stata Corp, College Station, Texas).

## Results

### Study population

79 patients underwent lung transplant during the 3.1 year study enrollment period, of whom 54 (68.3%) underwent telomere analysis of explanted lung tissue. There was no significant difference in age, sex, native lung disease, FEV1 prior to transplant, or LAS in patients who were and were not included. Donor lymphocyte tissue was available for 45 (83.3%) of the included recipients. There was no significant difference in donor age or recipient characteristics for those for whom donor lymphocytes were and were not available. Demographic and clinical characteristics of the patients included in the cohort are in Table 1.

**Table 1:** Demographic and clinical characteristics of patients included in the study cohort.

Donor age (median, IQR), yr	34.0 (20.0–52.0)
Donor telomere length (mean T/S ± SD)	0.73 ± 0.16
Recipient age (median, IQR), yr	63.0 (56.5–67.0)
Recipient telomere length (mean T/S ± SD)	0.62 ± 0.25
Male sex, n (%)	36 (66.7)
Native disease pulmonary fibrosis, n (%)	39 (72.2)
Most recent percent predicted FEV1 prior to transplantation (mean ± SD)	40.8 ± 18.0
Lung allocation score at the time of transplantation (mean ± SD)	50.0 ± 15.3
Died during the study period, n (%)	13 (24.1)

FEV1, forced expiratory volume in 1 second; IQR, interquartile range; SD, standard deviation.

**Fig 1:**
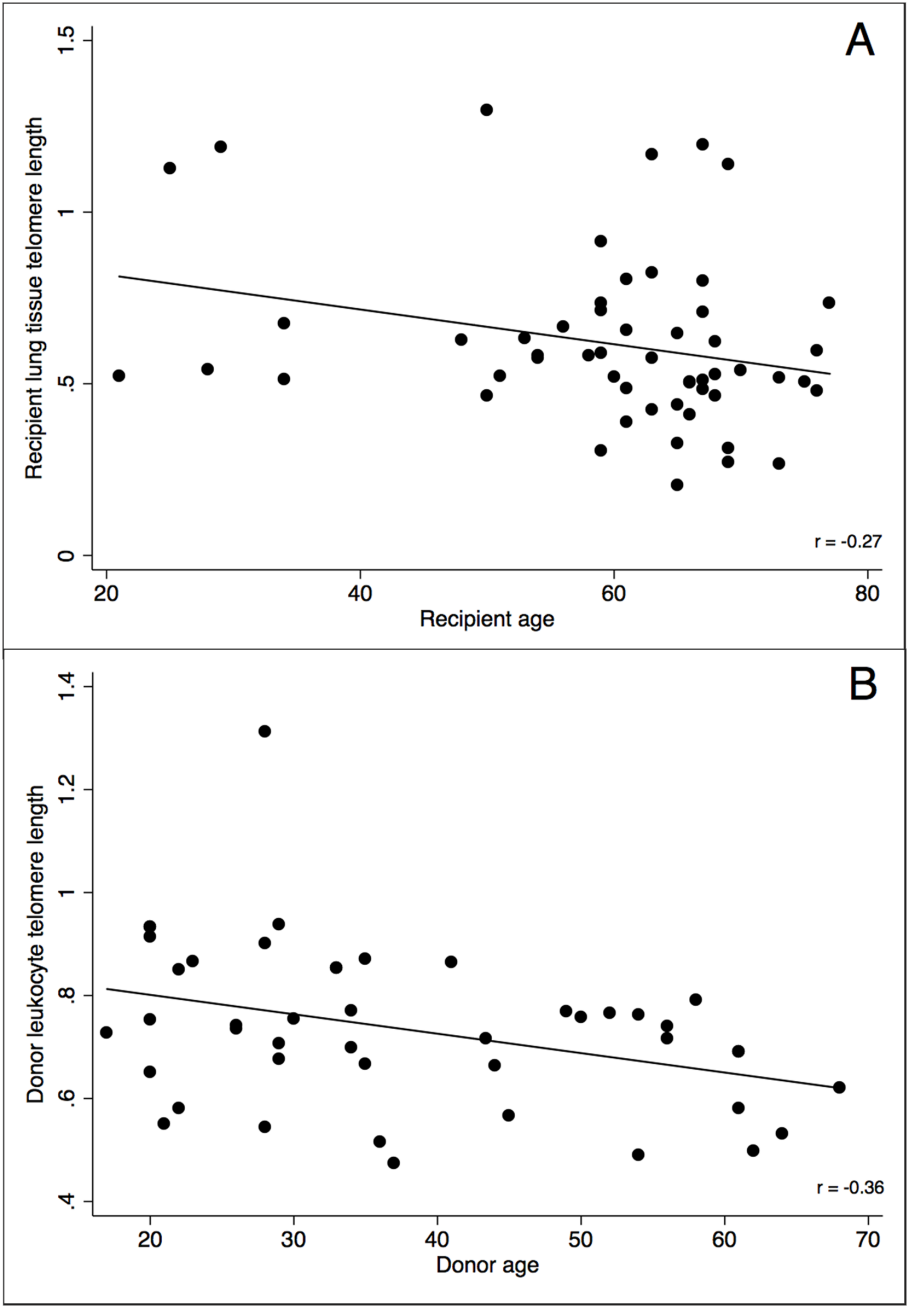
Scatterplot showing relationship between: A) recipient age and recipient telomere length (r = -0.26) (*P* = 0.05) and B) donor age and donor telomere length (r = -0.36) (*P* = 0.01).

The median donor telomere length was 0.74 (interquartile range (IQR) = 0.65–0.85). There were 3 donors (6.7%) with T/S ratio <0.50. The median recipient telomere length was 0.56 (IQR = 0.48–0.70). There were 15 recipients (27.8%) with a T/S ratio <0.50. There was no difference in telomere length in recipients who were and were not on chronic steroids—defined as >5 mg prednisone daily—prior to transplant, (0.63 ± 0.25 vs. 0.59 ± 0.25, p = 0.58), even after adjusting for age and native lung disease. There was an inverse correlation between telomere length and age in both recipients (r = -0.26, p = 0.05) and donors (r = -0.36, p = 0.01) (Fig 1). Donor lymphocyte telomeres were significantly longer than recipient lung tissue telomeres (mean T/S 0.73 ± 0.16 vs. 0.62 ± 0.25, p = 0.008).

We classified donor telomere lengths into those with long (third tertile) and short (other tertiles) telomeres as previously described [9,14]. We used an analogous classifier for the recipient lung telomere lengths, into short (first tertile) and long (other tertiles). Donors in the longest tertile of telomere length had a T/S > 0.77. Recipients in the shortest tertile of telomere length had a T/S < 0.51.

### Donor and recipient telomere length and survival

The mean length of follow-up from transplantation was 2.1 years. Long donor lymphocyte telomeres were not associated with survival following lung transplant, neither in unadjusted models (hazard ratio [HR] = 0.35, 95% confidence interval (CI) = 0.08–1.58, p = 0.17) nor when adjusted for donor and recipient age, native lung disease, LAS, and percent predict FEV1 (HR = 0.58, 95% CI = 0.12–2.85, p = 0.50) (Table 2). Similarly, recipient telomere length was not associated with survival (unadjusted HR = 0.51, 95% CI = 0.14–1.90, p = 0.32); (adjusted HR = 1.01, 95% CI = 0.50–2.05, p = 0.96) (Table 3). Neither donor nor recipient telomere length, when considered as a continuous variable, was associated with adjusted survival.

**Table 2:** Adjusted association between donor lymphocyte telomere length and survival following lung transplantation.

Characteristic	Hazard Ratio (95% CI)	P-value
Long donor telomeres	0.58 (0.12–2.85)	0.50
Donor age	1.05 (1.00–1.10)	0.05
Recipient age	0.98 (0.93–1.04)	0.57
LAS	1.02 (0.97–1.07)	0.42
FEV1 prior to transplant	0.03 (<0.01–4.95)	0.17
Native disease pulmonary fibrosis	1.26 (0.19–8.41)	0.81

CI, confidence interval; FEV1, forced expiratory volume in 1 second; LAS, lung allocation score

**Table 3:** Adjusted association between recipient lung tissue telomere length and survival following lung transplantation.

Characteristic	Hazard Ratio (95% CI)	P-value
Short recipient telomeres	1.01 (0.50–2.05)	0.97
Donor age	1.04 (1.00–1.09)	0.04
Recipient age	1.00 (0.95–1.06)	0.87
LAS	1.01 (0.96–1.05)	0.71
FEV1 prior to transplant	0.01 (<0.01–1.73)	0.08
Native disease pulmonary fibrosis	1.59 (0.27–9.15)	0.61

CI, confidence interval; FEV1, forced expiratory volume in 1 second; LAS, lung allocation score

### Recipient telomere length and secondary post-transplantation outcomes

Short recipient telomere length was not associated with leukopenia requiring GCSF or cessation of immunosuppression/antiviral medication, liver injury, acute kidney injury, kidney injury requiring dialysis, or CMV reactivation after stopping antivirals (Table 4). Recipients with short telomeres were less likely to have ACR in the first year following transplant (23.5% vs. 58.8%, p = 0.02). Long donor telomere length was not associated with ACR (53.3% vs. 40.7%, p = 0.52).

**Table 4:** Association of short recipient lung tissue telomere length and secondary post-transplantation outcomes.

Outcome	Other recipient telomere length (n = 36)	Short recipient telomere length (n = 18)	P-Value
Leukopenia requiring GCSF or cessation of immunosuppression/antiviral medication, n (%)	18 (50.0)	6 (33.3)	0.38
Transaminitis > 3x upper limit of normal, n (%)	7 (19.4)	4 (22.2)	1.00
Acute kidney injury, n (%)	22 (61.1)	12 (66.7)	0.77
Kidney injury requiring dialysis, n (%)	4 (11.1)	4 (22.2)	0.42
CMV reactivation after stopping antivirals, n (%)^a^	5 (15.6)	3 (18.8)	1.00
Acute cellular rejection in the first year, n (%)^b^	20 (58.8)	4 (23.5)	0.02

CMV, cytomegalovirus; GCSF, granulocyte colony stimulating factor

^a^ out of 32 and 16 patients, respectively, who stopped antivirals

^b^ out of 34 and 17 patients, respectively, who had biopsies following transplantation

## Discussion

Telomere shortening has been described in all the advanced lung diseases—pulmonary fibrosis, bronchiectasis, and emphysema—represented in our cohort [4, 16, 17]. Donor and recipient telomere length in lung transplantation, however, has not been well characterized aside from patients with known short telomere syndrome. Our primary findings were that both donor lymphocyte and recipient lung telomere lengths were inversely related to age and that there was a wide range of telomere lengths in both donors and recipients. Our secondary findings were that neither long donor lymphocyte telomeres nor short recipient lung tissue telomere length is associated with survival following lung transplantation. Short recipient lung telomere length is associated with decreased rate of acute cellular rejection but not with extra-pulmonary organ dysfunction.

The inverse correlation between recipient and donor telomere length and age was consistent with prior studies in the HCT literature. For example, Gadalla et al found similar correlation coefficients in their cohort (r = -0.20 and r = -0.31, for donors and recipients, respectively) [10]. They also found similar T/S ratio cutoffs for long donors, when dividing their cohort into tertiles (T/S >0.81 vs. >0.77 in the current study). The T/S ratio cutoff for short recipients in our cohort was below that reported in Gadalla et al (T/S<0.62 vs. 0.51 in the current study). This likely reflects the fact that we measured telomere length in recipient lung tissue whereas telomere length in recipient leukocytes was utilized in the aplastic anemia study. We note that the T/S ratio of <0.51 was also significantly lower than mean and lowest tertile T/S ratios reported in larger population-based studies [14].

Our decision to measure telomere length in recipient explant lung tissue was partly a matter of necessity, as we had limited access to recipient leukocytes before transplant and measurement of leukocyte telomeres post-transplant may be confounded by immunosuppressive medications[18]. Nevertheless, it is plausible that particularly short lung telomeres in the setting of advanced lung disease would be a marker of poor replicative reserve in other organs. This would place shorter telomere recipients at risk for worse survival and for extra-pulmonary organ dysfunction. We did not, however, find higher rates of renal failure requiring dialysis, significant liver injury, or need for modification of immunosuppression in recipients with shorter lung tissue telomeres. We did find lower rates of acute rejection in recipients with shorter telomeres, which is consistent with a case report of a patient with short telomere syndrome who did not develop rejection despite reduction in immunosuppressive medications[19]. It is also consistent with lower rates of pulmonary rejection in children who had lung transplantation following HCT[20]. In both populations, the presumed mechanism was related to reduced immunologic reserve related to telomere shortening.

The lack of association between recipient lung telomeres and survival or extra-pulmonary outcomes may be because of poor correlation between recipient lung telomeres and telomere length and cellular replicative reserve in other organs. Our study was underpowered to detect small or moderate effect sizes and it is possible that larger cohort would identify a relationship that we did not detect. Previous studies of recipient leukocyte telomere length in HCT, however, have not shown an impact on survival, which is consistent with our findings[21]. It may be that, even with relatively short telomeres, lung transplant recipients with shorter telomeres still had sufficient replicative reserve to limit the impact of short telomeres on post-transplant outcomes.

Increased donor age is associated with worse survival following lung transplant in some but not all studies [22, 23]. In our cohort, this relationship persisted even after adjusting for donor telomere length, suggesting that the negative impact of donor age was not merely of function of cellular replicative reserve. The lack of association between donor telomere length and survival is somewhat surprising since donor telomere length was strongly associated with survival in HCT recipients [9]. Given our limited follow-up time and the relatively limited sample size, we may not have accrued enough events to detect a difference, although there was little suggestion of a survival benefit in the current analysis. It may be that longer donor telomeres are predictive of graft function as has been suggested in renal transplant[24]. Alternatively, it may be that the rate of telomere shortening rather than the starting telomere length is more relevant to graft function, as has been suggested in liver transplant[25].

Our study has several limitations. First, although this is the first study to report specifically on recipient lung tissue telomere length, this was a relatively small cohort with a limited follow-up period. Second, we do not know to what extent recipient donor lymphocyte telomere length correlated with lung telomere length. Among older patients, there is little correlation between leukocyte telomere length and telomere length in other tissues, but it is unknown whether this is true for donors in the age range in our cohort[26]. Measurement of donor lung telomere length, for example, on transbronchial biopsy tissue, would allow for direct comparison of donor lung tissue telomere length and survival. Third, we did not perform genome analysis on recipients to determine to what extent patients with short telomeres had known telomerase mutations or whether such patients may have had a disproportionate benefit from longer donor telomeres. Finally, we did not have a significant number of donors or recipients at the extremes of telomere length. We do not know whether recipient survival is worse in patients with extremely short lung tissue telomeres or whether survival is improved in patients whose donors have extremely long telomeres.

## Conclusion

In this small observational cohort study, a significant portion of donors and recipients had relatively short telomeres in lymphocytes and lung tissue, respectively. Neither long donor telomeres nor short recipient telomeres, however, were associated with adjusted survival after lung transplantation. Short recipient telomeres were associated with decreased rates of acute cellular rejection but not extra-pulmonary organ dysfunction after transplantation. Larger cohort studies are needed to confirm these findings.
